# Maternal nematode infection upregulates expression of Th2/Treg and diapedesis related genes in the neonatal brain

**DOI:** 10.1038/s41598-021-01510-0

**Published:** 2021-11-11

**Authors:** Nawal El Ahdab, Manjurul Haque, Ejimedo Madogwe, Kristine G. Koski, Marilyn E. Scott

**Affiliations:** 1 Institute of Parasitology, McGill University (Macdonald Campus), 21,111 Lakeshore Road, Ste-Anne de Bellevue, QC H9X 3V9 Canada; 2 Department of Animal Science, McGill University (Macdonald Campus), 21,111 Lakeshore Road, Ste-Anne de Bellevue, QC H9X 3V9 Canada; 3 School of Human Nutrition, McGill University (Macdonald Campus), 21,111 Lakeshore Road, Ste-Anne de Bellevue, QC H9X 3V9 Canada

**Keywords:** Developmental biology, Immunology, Neuroscience, Zoology

## Abstract

Intestinal nematode infections common during pregnancy have recently been shown to have impacts that extend to their uninfected offspring including altered brain gene expression. If maternal immune signals reach the neonatal brain, they might alter neuroimmune development. We explored expression of genes associated with four distinct types of T cells (Th1, Th2, Th17, Treg) and with leukocyte transendothelial migration and endocytosis transport across the blood–brain barrier (BBB) in the postnatal brain of offspring of nematode-infected mice, through secondary analysis of a whole brain gene expression database. Th1/Th17 expression was lowered by maternal infection as evidenced by down-regulated expression of IL1β, Th1 receptors and related proteins, and of IL22 and several Th17 genes associated with immunopathology. In contrast, Th2/Treg related pathways were upregulated as shown by higher expression of IL4 and TGF-β family genes. Maternal infection also upregulated expression of pathways and integrin genes involved in transport of leukocytes in between endothelial cells but downregulated endosome vesicle formation related genes that are necessary for endocytosis of immunoglobulins across the BBB. Taken together, pup brain gene expression indicates that maternal nematode infection enhanced movement of leukocytes across the neonatal BBB and promoted a Th2/Treg environment that presumably minimizes the proinflammatory Th1 response in the pup brain.

## Introduction

Chronic gastrointestinal (GI) nematode infections are extremely important in low-income countries where human hookworm infections exacerbate anemia during pregnancy^1^ and in ruminants where GI nematodes lower birth weight^2^. On the other hand, GI nematode infections have been observed to enhance immune development in mouse^3,4^ and human^5^ studies and enhance immunity against non-infectious Th2-related conditions in human^6,7^ and mouse^8^ neonates, at least in part through transfer of cytokines and immunoglobulins through breast milk. Moreover, enhanced neonatal systemic immunity in response to maternal GI nematodes has been shown to promote long-lasting immunity against nematode infection in the offspring^4^. Thus, GI nematodes might have both harmful and beneficial consequences for the next generation, and benefits might be a consequence of the Th2/Treg responses typically induced by GI nematodes that dampen Th1/Th17 immunopathology^9,10^.

Infection of pregnant and lactating mice with the GI nematode *Heligmosomoides bakeri* (= *Heligmosomoides polygyrus, Nematospiroides dubius*) has been shown to alter brain gene expression in the late-term fetus^11^ and the 7-day old (P7) neonates^12^ relative to offspring of uninfected mothers. Particularly intriguing was the upregulation of long-term potentiation (LTP) and related pathways in the P7 pup brain. LTP promotes synaptogenesis, spatial learning and memory^13–15^ and is observed when the neonatal brain is exposed to Th2 conditions^16^ but impaired in IL4 knock-out mice^17^. Thus, the upregulated expression of LTP in response to maternal *H. bakeri* infection not only indicated a possible benefit for the pups of infected dams but also raised the possibility that the well-documented Th2/Treg response to *H. bakeri* in the infected host^18^ might be reflected in the brains of their uninfected pups.

Endocytosis^19^ and transendothelial leukocyte migration^20^ allow immune elements such as antigens, immunoglobulins, cytokines, and leukocytes obtained from breast milk^21^ or produced by the neonate to cross the blood–brain barrier (BBB). Cytokines^22^ and immunoglobulins^23^ bind to surface receptors on endothelial cells and the resulting complex triggers an endocytosis signalling cascade that induces vesicle formation, budding, and intracellular transport^24^. Although leukocytes might also enter the brain through endocytosis, activated leukocytes more commonly enter between endothelial cells through diapedesis^20^. Proteins such as integrins on the surface of leukocytes dock with cell adhesion molecules (CAMs) and other proteins on the surface of endothelial cells^25^. The resulting movement of the actin cytoskeleton^26^ inside endothelial cells loosens the tight junctions, allowing leukocytes to squeeze between endothelial cells^27^ and enter the brain. This process temporarily compromises junction integrity^27^ after which junction gene expression is upregulated to restore endothelial cell adherence and re-establish normal BBB integrity^28^.

Given that *H. bakeri* induces a Th2/Treg response in the infected host^18^, that maternal immune elements are transferred to the neonate, and that Th2 responses favour LTP, we hypothesized that neonatal brain gene expression in response to maternal infection might reflect a heightened Th2/Treg profile and dampened Th1/Th17 associated neuro-inflammation, especially if movement of either leukocytes or immunoglobulins across the BBB was compromised. The goal of this secondary analysis was to explore an existing brain gene expression database for evidence that maternal *H. bakeri* infection altered the profile of T helper cell responses in the P7 brain. The first specific objective was to determine whether or not expression of genes related to innate and/or adaptive immune responses was shifted toward a Th2/Treg profile. The second objective was to determine whether or not maternal infection altered expression of genes involved in transport of immune elements across the BBB, with a focus on endocytosis and associated vesicle transport of immunoglobulins, and on transendothelial migration and regulation of junctions involved in transport of leukocytes. Rather than relying on pathway analysis, we manually explored context relevant cascades within pathways by inspecting expression data for ligands and receptors to assess pathway activation and by inspecting products to assess function. This allowed us to identify differentially expressed sets of genes that would be expected to alter function.

## Results

### Impact of Maternal Infection on Immune-Related Genes in the Neonatal Brain

Differential expression of immune related pup brain genes was examined for evidence that maternal intestinal nematode infection might have altered components of the innate immune system and/or the adaptive immune system. Given that *H. bakeri* infection typically upregulates Th2/Treg responses and downregulates Th1/Th17 responses, genes related to these responses were of particular interest.

### Limited negative impact on expression of innate immune genes

The innate immune response to pathogens involves the hematopoietic cell lineage, the complement and coagulation cascade and platelet activation pathways as well as several receptor-mediated (Toll, Imd, NOD and RIG-like) signalling pathways that recognize molecular signals of pathogens and activate an adaptive response. Based on the previous KEGG pathway analysis^12^, only the RIG-I like receptor signalling KEGG pathway (Table 1) that activates an innate immune response to viral pathogens was downregulated. We also observed downregulated expression of seven of the eight differentially expressed cell surface markers and all nine of the differentially expressed chemokine ligand genes involved in the myeloid cell lineage that generates innate immune cells (Table 2). Thus, the maternal infection had a modest negative impact on the neonatal brain innate immune system.

**Table 1:** List of immune related KEGG pathways considered in this study.

Classification	Pathway name	Differential expression^1^	Reference number
General immunity	Hematopoietic cell lineage	N/A	04640
	Cytosolic DNA sensing	N/A	04623
	Intestinal immune network for IgA production	Downregulated	04672
Innate immunity	Fc gamma R mediated phagocytosis	Upregulated	04666
	Antigen processing and presentation	N/A	04612
	Complement and coagulation cascade	N/A	04610
	Platelet activation	N/A	04611
	Toll-like receptor signaling	N/A	04620
	Toll and Imd signaling	N/A	04624
	NOD-like receptor signaling	N/A	04621
	RIG-I-like receptor signaling	Downregulated	04622
Adaptive immunity	Chemokine signaling	Upregulated	04062
	Cytokine-cytokine receptor interaction	Downregulated	04060
	Fc epsilon RI signaling	Upregulated	04664
	C-type lectin receptor signaling – polarize T cell responses	N/A	04625
	Natural killer cell mediated cytotoxicity / adaptive induces apoptosis	N/A	04650
	T cell receptor signaling	Upregulated	04660
	B cell receptor signaling	Upregulated	04662
	Th1 and Th2 cell differentiation	N/A	04658
	Th17 cell differentiation	N/A	04659
	IL-17 signaling	N/A	04657
BBB related	Leukocyte transendothelial migration	Upregulated	04670
	Tight junction	Upregulated	04530
	Adheren junction	Upregulated	04520
	Endocytosis	Upregulated	04144
	Regulation of actin cytoskeleton^2^	Upregulated	04810

^1^Differential regulation as reported by Haque et al.^12^.

^2^Subpathway of the Leukocyte transendothelial migration pathway.

**Table 2:** List of innate immune system related genes differentially expressed in the pup brain, in response to maternal *H. bakeri* infection.

Classification	Gene name	Gene symbol	*p* value	Log 2 fold change
CD cell surface markers	CD302 antigen	*CD302*	7.17E−12	− 1.8988
	CD40 antigen	*CD40*	4.33E−10	− 1.7484
	CD83 antigen	*CD83*	3.03E−08	− 1.2487
	CD300A antigen	*CD300A*	4.65E−08	− 1.4979
	CD209f antigen	*CD209F*	9.01E−07	− 1.4813
	CD93 antigen	*CD93*	1.05E−05	1.3519
	CD200 receptor 1	*CD200R1*	1.99E−05	− 1.5711
	CD209g antigen	*CD209G*	3.77E−05	− 1.9848
Chemokines	chemokine (C–C motif) ligand 9	*CCL9*	8.11E−13	− 2.2198
	chemokine (C–C motif) ligand 6	*CCL6*	1.49E−10	− 1.9152
	chemokine (C–C motif) receptor 1	*CCR1*	6.32E−08	− 1.5864
	chemokine (C–X–C motif) ligand 1	*CXCL1*	5.24E−08	− 1.9013
	chemokine (C–C motif) ligand 25	*CCL25*	1.74E−08	− 1.6614
	chemokine (C–C motif) ligand 12	*CCL12*	7.17E−07	− 1.9695
	chemokine-like factor	*CKLF*	3.37E−06	− 1.2909
	chemokine (C–C motif) ligand 24	*CCL24*	9.86E−05	− 1.2683
	chemokine (C–C motif) ligand 7	*CCL7*	3.00E−05	− 1.6164

Differential regulation as reported by Haque et al.^12^.

### Differential expression of adaptive immune genes

Consistent with upregulation of KEGG pathway maps for T cell receptor signaling and B cell receptor signaling (Table 1;^12^), many pup brain genes of the adaptive immune system were differentially expressed. Among those associated with leukocyte, lymphocyte, and immunoglobulin superfamilies (Table 3), expression of four nuclear factors of activated T cells (*NFAT5, NFATC1, NFATC2, NFATC3*), a T cell transcription factor (*TCF7L1*), and early B cell factor 3 (*EBF3*) was upregulated (Table 3) and expression of leukocyte transcript (*LST1*), lymphocyte antigens (*LY86, LY6G6D*), cytotoxic T-lymphocyte associated proteins (*CTLA2A, CTLA2B*), T cell proliferation (*MTCP1*), T cell linkers of activation (*LAT, LAT2*) and a B cell receptor associated protein (*BCAP29*) was downregulated. Within the immunoglobulin superfamily, *IGSF3* was upregulated and *GM4926* expression was downregulated. All differentially expressed CD cell surface markers and 5 of 6 chemokines were downregulated (Table 3). Taken together, these results highlight the responsiveness of adaptive immune genes in the pup brain to maternal nematode infection.

**Table 3:** List of adaptive immune system related genes differentially expressed in the pup brain, in response to maternal *H. bakeri* infection.

Classification	Gene name	Gene symbol	*p *value	Log 2 Fold change
Leukocytes	Leukocyte specific transcript 1	*LST1*	3.30E−08	− 1.4504
Lymphocytes	Lymphocyte antigen 86	*LY86*	1.55E−08	− 1.6468
	Lymphocyte antigen 6 complex, locus G6D	*LY6G6D*	7.51E−05	− 2.0459
	Lymphocyte protein tyrosine kinase	*LCK*	3.31E−10	− 1.2848
	Cytotoxic T lymphocyte-associated protein 2 alpha	*CTLA2Α*	1.71E−11	− 1.9618
	Cytotoxic T lymphocyte-associated protein 2 beta	*CTLA2Β*	3.60E−09	− 2.041
	Transcription factor 7 like 1 (T cell specific, HMG box)	*TCF7L1*	3.83E−07	1.5258
	Mature T cell proliferation 1	*MTCP1*	7.32E−07	− 1.3497
	Linker for activation of T cells family, member 2	*LAT2*	3.64E−06	− 1.9848
	Linker for activation of T cells	*LAT*	2.73E−05	− 1.9806
	Nuclear factor of activated T cells, cytoplasmic, calcineurin dependent 1	*NFATC1*	9.49E−07	1.3363
	Nuclear factor of activated T cells, cytoplasmic, calcineurin dependent 2	*NFATC2*	1.11E−05	1.2136
	Nuclear factor of activated T cells, cytoplasmic, calcineurin dependent 3	*NFATC3*	6.84E−09	1.2199
	Nuclear factor of activated T cells 5	*NFAT5*	1.68E−14	2.378
	X-linked lymphocyte-regulated complex	*XLR*	1.26E−06	− 1.7171
B cells	Early B cell factor 3	*EBF3*	3.53E−15	1.6944
	B cell receptor associated protein 29	*BCAP29*	3.70E−09	− 1.4942
Immunoglobulin superfamily	Immunoglobulin superfamily, member 3	*IGSF3*	2.69E−08	1.7284
	T-cell immunoglobulin and mucin domain containing 2 pseudogene	*GM4926*	1.69E−06	− 1.5855
CD cell surface markers	CD1d1 antigen	*CD1D1*	1.60E−05	− 1.2071
	CD48 antigen	*CD48*	5.13E−07	− 1.8838
	CD52 antigen	*CD52*	1.50E−05	− 1.7423
	CD53 antigen	*CD53*	1.94E−10	− 1.5542
	CD59a antigen	*CD59A*	4.72E−08	− 1.7165
	CD63 antigen	*CD63*	9.54E−08	− 1.4596
	CD84 antigen	*CD84*	6.02E−07	− 1.4359
	CD86 antigen	*CD86*	2.09E−07	− 1.4856
	CD320 antigen	*CD320*	3.60E−11	− 1.3617
Chemokine	Chemokine (C–X–C motif) ligand 11	*CXCL11*	4.45E−05	− 1.7877
	Chemokine (C–C motif) ligand 24	*CCL24*	9.86E−05	− 1.2683
	Chemokine (C–C motif) ligand 25	*CCL25*	1.74E−08	− 1.6614
	Chemokine (C–C motif) ligand 27A	*CCL27A*	4.27E−09	− 1.8182
	Chemokine (C–X–C motif) receptor 5	*CXCR5*	6.26E−05	1.7003
	Chemokine-like factor 1	*CKLF*	3.37E−06	− 1.2909

Differential regulation as reported by Haque et al.^12^.

### Downregulated Th1/Th17 gene expression

The original KEGG pathway analysis^12^ revealed that the Th1 and Th2 cell differentiation pathway was not differentially regulated (Table 1) but, as this pathway generates both Th1 and Th2 responses, our in-depth exploration of gene expression revealed several intriguing results (Table 4). We observed upregulated expression of the intermediate complex *MAML1* (Supplementary Table 1) and receptor *IL12RB2* both of which activate Th1 cell differentiation. Although this hints at a heightened Th1 response, expression of four Th1 interleukins (*IL1B, IL15, IL15RA, IL18*) was downregulated. In addition, one TNF superfamily alpha inducible gene (*TNFAIP8L2*), one TNF superfamily receptor (*TNFRSF12A),* three INF inducible or induced proteins (*IFI27L2A, IFI47, IFI35*) and one INF stimulated protein (*ISG20*) were also downregulated (Table 4). Furthermore, in depth analysis of the Th17 signalling KEGG pathway revealed downregulation of its product (*IL22*) and four genes associated with immunopathology (*CCL7, S100A8, S100A9*, *MMP13*) (Table 4) but upregulation of *IL17RD,* a negative regulator of inflammation^29^. Together, these observations indicate that maternal *H. bakeri* infection might have limited Th1/Th17 inflammation and immunopathology.

**Table 4:** List of differentially expressed cytokine related genes classified by immune response in the pup brain, in response to maternal *H. bakeri* infection.

Immune response	Classification	Gene name	Gene symbol	*p* value	Log 2 fold change
Th1	Interferon	Interferon, alpha-inducible protein 27 like 2A	*IFI27L2A*	2.51E−09	− 2.5516
		Interferon gamma inducible protein 47	*IFI47*	6.30E−07	− 1.6666
		Interferon-induced protein 35	*IFI35*	2.15E−06	− 1.3033
		Interferon-stimulated protein	*ISG20*	7.81E−07	− 1.442
	Tumor necrosis factor	Tumor necrosis factor, alpha-induced protein 8-like 2	*TNFAIP8L2*	5.60E−08	− 1.6267
		Tumor necrosis factor receptor superfamily, member 11a	*TNFRSF11A*	2.39E−08	1.2215
		Tumor necrosis factor receptor superfamily, member 12a	*TNFRSF12A*	6.00E−06	− 1.2305
	Interleukin	Interleukin 1 beta	*IL1B*	1.33E−07	− 3.1953
		Interleukin-1 receptor-associated kinase 1 binding protein 1	*IRAK1BP1*	3.38E−10	− 1.5769
		Interleukin 15	*IL15*	6.30E−06	− 1.662
		Interleukin 15 receptor, alpha chain	*IL15RA*	4.79E−05	− 1.3734
		Interleukin 18	*IL18*	4.49E−09	− 1.4295
		Interleukin 12 receptor, beta 2	*IL12RB2*	1.32E−06	2.0879
Th2	Interleukin	Interleukin 4	*IL4*	1.81E−07	1.2171
		Interleukin 13 receptor, alpha 2	*IL13RA2*	1.62E−09	− 1.9619
		Interleukin enhancer binding factor 2	*ILF2*	6.79E−08	− 1.2226
Treg	Transforming growth factor	Transforming growth factor, beta 2	*TGFB2*	2.55E−07	1.0248^1^
		Transforming growth factor, beta receptor III	*TGFBR3*	4.69E−08	1.2645
		Transforming growth factor, beta receptor associated protein 1	*TGFBRAP1*	8.92E−07	1.3063
		Latent transforming growth factor beta binding protein 3	*LTBP3*	1.35E−07	1.3637
		Latent transforming growth factor beta binding protein 4	*LTBP4*	3.43E−06	1.6984
		Transforming growth factor alpha	*TGFA*	1.30E−06	1.2669
	Interleukin	Interleukin 10-related T cell-derived inducible factor beta	*ILTIFB*	3.20E−07	− 2.3961
Th17	Interleukin	Interleukin 17 receptor D	*IL17RD*	4.41E−10	1.9374
		Interleukin 22	*IL*22	9.41E−06	− 2.162
	Chemokine	Chemokine (C–C motif) ligand 7	*CCL7*	3.00E−05	− 1.6164
	Related proteins	S100 calcium binding protein A8 (calgranulin A)	*S100A8*	3.07E−08	− 2.4356
		S100 calcium binding protein A9 (calgranulin B)	*S100A9*	2.39E−07	− 2.0865
		Matrix metallopeptidase 13	*MMP13*	4.26E−05	− 1.7194

Differential regulation as reported by Haque et al.^12^.

^1^Borderline significant value.

### Upregulated Th2/Treg gene expression

As expected given the cross-regulation between Th1/Th17 and Th2/Treg responses, maternal *H. bakeri* infection upregulated Th2/Treg gene expression in the pup brain, as evidenced by the relatively consistent pattern of upregulation from receptor (*Notch1/2*) to its intermediate complex *MAML1* (see Supplementary Table 1) to *IL4* (Table 4), the hallmark Th2 product of the Th1and Th2 cell differentiation pathway. Among Treg-related genes (Table 4), expression of TGF-β receptor 3 (*TGFBR3*), TGF-β receptor-associated protein (*TGFBRAP1*), latent TGF-β binding proteins (*LTBP3, LTBP4*) and *TGFA* were upregulated, and *TGFB2* expression was upregulated in response to maternal infection although it did not meet our log 2 fold change cut-off. These findings are consistent with a dominant Th2/Treg bias in response to maternal *H. bakeri* infection, a response that might play an important role in modulating inflammation and auto-immune responses in the brains of the uninfected neonates.

### Impact of Maternal Infection on Genes Involved in Transport of Immune Signals across the BBB

Based on our previous KEGG pathway analysis^12^, there was evidence that maternal *H. bakeri* infection altered the expression of several pathways involved in transport of immune signals across the BBB. Five pathways (transendothelial migration, regulation of actin cytoskeleton, adheren junction, tight junction, endocytosis) were upregulated whereas two pathways (cytokine-cytokine receptor interaction, soluble *N*- ethylmaleimide-sensitive factor attachment protein receptor (SNARE) interactions for vesicular transport) were downregulated (Table 1)^12^. To gain better clarity, gene expression data were probed to more precisely define functions by which a maternal nematode infection might have altered transendothelial migration of leukocytes and receptor-mediated endocytosis of cytokines and immunoglobulins.

### Heightened leukocyte transendothelial cell migration

Leukocyte migration involves integrins that allow docking and diapedesis and the dynamically responsive actin cytoskeleton that allows endothelial cells to expand and contract as leukocytes pass in between them. Expression of three integrin alpha genes (*ITGA3,4,11*) and integrin beta (*ITGB4*) was upregulated, and expression of integrin beta 1 binding protein 1 (*ITGB1BP1*) was downregulated by maternal infection (Table 5). Among the matrix metallopeptidases (*MMPs*) that are regulated by actin cytoskeleton remodeling and involved in formation of transcellular channels, maternal *H. bakeri* infection upregulated *MMMP15* and downregulated *MMP13* (Table 5).

**Table 5:** List of cell adhesion molecules and related genes involved in leukocyte transendothelial cell migration that are differentially expressed in the pup brain, in response to maternal *H. bakeri* infection.

Classification	Gene name	Gene symbol	*p* value	Log 2 fold change
Leukocyte transendothelial cell migration	Integrin alpha 3	*ITGΑ3*	1.16E−06	1.2366
	Integrin alpha 4	*ITGΑ4*	3.81E−08	1.2737
	Integrin alpha 11	*ITGΑ11*	2.20E−08	1.7196
	Integrin alpha E, epithelial-associated	*ITGΑE*	3.47E−06	− 2.3048
	Integrin beta 4	*ITGΒ4*	1.71E−09	1.8016
	Integrin beta 1 binding protein 1	*ITGΒ1BP1*	6.09E−11	− 1.9182
	Calcium and integrin binding 1 (calmyrin)	*CIB1*	1.04E−09	− 1.4908
	Calcium and integrin binding family member 2	*CIB2*	6.26E−09	− 1.3909
	Matrix metallopeptidase 13	*MMP13*	4.26E−05	− 1.7194
	Matrix metallopeptidase 15	*MMP15*	4.67E−05	1.295
Adheren junctions	Cadherin 3	*CDH3*	1.01E−08	1.6134
	Cadherin 4	*CDH4*	4.83E−06	1.3775
	Cadherin 5	*CDH5*	2.41E−05	1.2137
	Cadherin 6	*CDH6*	5.87E−07	1.6177
	Cadherin 23 (otocadherin)	*CDH23*	8.92E−09	1.9526
	Cadherin, EGF LAG seven-pass G-type receptor 1	*CELSR1*	2.34E−11	2.5308
	Cadherin, EGF LAG seven-pass G-type receptor 2	*CELSR2*	2.81E−08	2.8609
	Cadherin, EGF LAG seven-pass G-type receptor 3	*CELSR3*	5.52E−10	2.3788
	Catenin (cadherin associated protein), delta 2	*CTNND2*	7.83E−07	1.5838
	Catenin (cadherin associated protein), delta 1	*CTNND1*	2.55E−06	1.2385
	Desmoglein 2	*DSG2*	2.09E−05	1.2256
Tight junctions	Vinculin	*VCL*	2.16E−07	1.7986
	Tight junction protein 1	*TJP1*	9.28E−09	1.234
	Cingulin	*CGN*	1.11E−05	1.3057
	Cingulin-like 1	*CGNL1*	8.38E−11	1.9121
	Occludin/ELL domain containing 1	*OCEL1*	1.48E−09	− 1.2976
	Claudin 10	*CLDN10*	3.11E−09	− 1.5952

Differential regulation as reported by Haque et al.^12^. Differentially expressed genes related to cell adhesion, cell migration, and junction units that are independent of leukocyte transendothelial migration are shown in Supplementary Table 1.

Transport of leukocytes between endothelial cells is regulated by adheren junctions, tight junctions and gap junctions. Among the genes involved in adheren junctions, maternal infection upregulated expression of eight cadherins (*CDH3,4,5,6,23; CELSR1,2*), two catenins (*CTNND1,* 2) and desmogliein (*DSG2*) (Table 5) providing a strong indication that the function of these junctions was heightened in response to a maternal nematode infection. With respect to tight junctions, expression of vinculin (*VCL)*, tight junction protein 1 (*TJP1*) and two cingulins (*CGN, CGNL1*) involved in actin binding was upregulated whereas expression of genes associated with sealing tight junctions (the occludin, *OCEL1* and the claudin, *CLDN10*) was downregulated (Table 5). Of note, we found no evidence of differential expression of genes associated with gap junctions.

Taken together, these gene expression data provide evidence of more dynamic interactions between junctions and the actin cytoskeleton at the BBB of neonates of infected mothers which would be consistent with heightened migration of leukocytes between endothelial cells.

### Endocytosis limited by impaired intracellular trafficking

Receptor-mediated endocytosis involves initiation and signalling as well as vesicle migration and endosome formation. We observed that maternal infection upregulated expression of eight genes involved in initiation and signalling including three involved in TGF-β transport (*TGFβ*, *TGFβR3*, *SMAD3*) as well as dynamin genes (*DNM3, DNMBP*) that are critical for vesicle budding, but downregulated expression of calveolin 2 (*CAV2*), a clathrin (*CLTA*) and epidermal growth factor receptor (*EGFR*) (Table 6). Importantly, however, we found evidence that intracellular trafficking was impaired based on downregulation of four sorting nexin family genes (*SNX1,2,5,7*), one vacuolar protein sorting gene (*VSP29*), one coiled-coil domain gene (*CCDC53*), and one charged multivesicular body protein gene (*CHMP2A*) that are all involved in vesicular migration and endosome formation. Among differentially expressed genes involved in intracellular trafficking, only the early endosome antigen 1 gene (*EEA1*) was upregulated by maternal *H. bakeri* infection (Table 6). Notably, the programmed cell death 6 (*PDCD6*) gene was downregulated as was the apoptosis pathway which suggests that maternal nematode infection might have downregulated apoptosis in the neonatal brain to further protect neural development.

**Table 6:** List of genes involved in endocytosis pathway that are differentially expressed in the pup brain, in response to maternal *H. bakeri* infection.

Classification	Gene name	Gene symbol	*p* value	Log 2 fold change
Initiation and signalling	Transforming growth factor, beta 2	*TGFB2*	2.55E−07	1.0248
	Transforming growth factor, beta receptor III	*TGFBR3*	4.69E−08	1.2645
	MAD homolog 3 (Drosophila)	*SMAD3*	6.35E−08	1.6784
	Dynamin 3	*DNM3*	4.95E−09	1.6835
	Dynamin binding protein	*DNMBP*	2.17E−05	1.5473
	Caveolin 2	*CAV2*	6.89E−07	− 1.2166
	Clathrin, light polypeptide (Lca)	*CLTA*	3.71E−08	− 1.2065
	EGF-like domain 7	*EGFL7*	5.75E−10	− 1.5489
	Epidermal growth factor receptor	*EGFR*	1.53E−11	1.4725
	Adaptor protein complex AP-2, alpha 1 subunit	*AP2A1*	6.49E−05	1.3822
	Protein kinase C, alpha	*PRKCA*	3.03E−07	1.5849
	Rous sarcoma oncogene	*SRC*	5.11E−06	1.462
Vesicle migration and endosome formation	Sorting nexin 1	*SNX1*	9.45E−08	− 1.1603
	Sorting nexin 2	*SNX2*	2.29E−07	− 1.1769
	Sorting nexin 5	*SNX5*	3.31E−07	− 1.234
	Sorting nexin 7	*SNX7*	1.98E−08	− 1.1221
	Vacuolar protein sorting 29 (S. pombe)	*VPS29*	2.19E−11	− 1.8892
	Coiled-coil domain containing 53	*CCDC53*	2.87E−09	− 1.4254
	Charged multivesicular body protein 2A	*CHMP2A*	5.63E−10	− 1.6867
	Early endosome antigen 1	*EEA1*	1.71E−07	1.2971
	RAB7, member RAS oncogene family-like 1	*RAB7L1*	1.71E−09	− 1.4527
	CDC42 binding protein kinase beta	*CDC42BPB*	7.17E−06	1.8055
	Programmed cell death 6	*PDCD6*	2.68E−10	− 1.7176
	Kinesin family member 5A	*KIF5A*	2.32E−07	1.6266
	ADP-ribosylation factor guanine nucleotide-exchange factor 2 (brefeldin A-inhibited)	*ARFGEF2*	1.21E−09	1.9556
	WAS protein family, member 2	*WASF2*	1.04E−07	1.5409

Differential regulation as reported by Haque et al.^12^.

Therefore, despite upregulation of the endocytosis KEGG pathway in the original study^12^, the observed downregulation of vesicle formation genes suggests that maternal infection might have impaired transport of immunoglobulins and cytokines across the BBB in the offspring of nematode infected dams.

### Confirmation of sequencing data by qPCR

Using qPCR, we validated the gene expression data reported in our RNA Hi-seq sequencing (Supplementary Table 2). Interestingly two of the upregulated genes reported by RNA seq data showed a higher fold-change in qPCR analysis than RNA seq (*TGFB2:* 3.1 *vs* 1.02 and *ITGA11*: 2.3 *vs* 1.71) in brains of pups of infected compared with uninfected dams. In addition, *VPS29* which was reportedly downregulated in RNA seq data showed a tendency to be lower in response to maternal nematode infection (*p* = 0.15).

## Discussion

Our comprehensive interrogation of KEGG pathway-associated genes in our list of pup brain genes that were differentially expressed genes in response to maternal *H. bakeri* infection revealed three key findings. Unlike many maternal stressors that are associated with neonatal neuro-inflammation^30–33^, we showed that maternal nematode infection downregulated expression of only a few cell surface markers and chemokine ligand genes indicating a very limited impact on innate immune genes. However, this maternal nematode infection restricted to the maternal intestine led to widespread differential expression of genes of the adaptive immune response in the neonatal brain. Most notable was the upregulation of genes related to Th2 and Treg responses and downregulation of genes related to Th1 and Th17 responses. This is consistent with the Th2/Treg response typical in the host infected with *H. bakeri*^18^. We also found a gene expression signature of heightened leukocyte migration between endothelial cells of the BBB in response to maternal nematode infection. The upregulated expression of genes involved in the leukocyte transendothelial migration pathway and in expression of integrins and other junction genes indicated enhanced migration of leukocytes which likely included Th2 and Treg cells into the neonatal brain. In contrast, lowered expression of genes needed for vesicular transport indicated impaired endocytosis of immune elements including cytokines and immunoglobulins. Taken together, these findings indicate a Th2/Treg biased response in the pup brain perhaps driven more by T cell entry in between endothelial cells of the BBB than by immunoglobulin or cytokine endocytosis.

Innate and adaptive immune responses play important homeostatic roles in the developing brain that promote neurodevelopment, limit neuro-inflammation and neurological diseases, and ensure that any pathogens that cross the BBB are efficiently recognized and controlled^34^. With respect to innate responses, in addition to our previous report^12^ of downregulated expression of the RIG-I-like receptor signaling KEGG pathway involved in recognition of viral pathogens^35^, we found that maternal infection downregulated expression of several chemokines and CD cell surface markers, suggesting a limited negative impact on innate immunity. However, in exploring genes associated with vesicle mediated transport, we also observed differential expression of several genes in a direction that suggested reduced programmed cell death. Though not a focus of this study, this latter observation raises the intriguing possibility that maternal nematode infection might limit apoptosis in the uninfected neonatal brain.

In contrast to the innate immune system, our analysis provided considerable evidence that maternal infection not only altered expression of the adaptive immune response but also led to Th2/Treg bias in the pup brain. Upregulated expression of B cell and T cell receptor signaling KEGG pathways was previously reported^12^ and our current study showed differential expression of numerous genes needed for Th1, Th2, Treg and Th17 responses including genes involved in T and B cell differentiation, maturation, migration, activation as well as receptors, ligands, and signalling molecules. These findings strongly indicate that maternal *H. bakeri* infection affected adaptive immunity in the uninfected pup brain. Furthermore, the upregulated expression of the hallmark Th2 cytokine *IL4*^36,37^ together with genes in its signaling cascade indicated a Th2 bias. As IL4 is an activator and recruiter of Th2 cells, downstream consequences might not yet be evident at P7, explaining why we did not detect differential expression of *IL13*, another hallmark Th2 cytokine^37^. In addition, the B cell receptor signaling pathway and the B cell development gene *EBF3* were upregulated. They are important in initiating a heightened Th2 cell response^38^. Consistent with an upregulated Th2 response, upregulated expression of TGF family genes including receptors, binding proteins, and receptor associated proteins all point to an upregulated Treg response. Together, these results clearly highlight that this maternal nematode infection shifted gene expression toward a Th2/Treg response in the brain of the uninfected neonate.

Further evidence of a Th2/Treg bias was seen in the dampened expression of genes involved in the Th1/Th17 responses, an observation that is consistent with the cross-regulation of these two arms of the adaptive immune system^10^. As IL4 and TGF-β both have a negative effect on Th1 cytokine production^9^, it was not surprising to see the downregulated expression of Th1 interleukins as well as *TNF* and *INF* related proteins in the neonatal brain. In addition, the autoimmune Th17 response was downregulated as indicated by the downregulation of its hallmark *IL22* coding gene as well as most autoimmune pathology genes in the IL17 signaling pathway.

The observed bias toward Th2/Treg adaptive immunity in response to *H. bakeri* is well documented in lymphoid tissues and blood of the infected mouse^27,40^ and there is evidence that a protective Th2 response against GI nematodes is transferred to the neonate through T cells and immunoglobulins in milk^4,39^. Transfer of *H. bakeri* specific IgG1 to the neonate has been shown to protect the pups from this infection^39^ and transfer of Th2 competent CD4 + T cells from mice infected with a related nematode (*Nippostrongylus brasiliensis*) has been shown to induce lasting protection against direct infection of the pup^4^. The maternal Th2/Treg bias extends beyond the intestine as seen in the lungs and spleen of neonates of *H. bakeri* infected mice^4^. Our results extend these systemic impacts of maternal nematode infection on immunity in the neonate to expression of the adaptive immune response in the pup brain.

We had hypothesized that the neonatal brain may have received signals of maternal infection through movement of leukocytes in between endothelial cells of the BBB. Based on our analysis of leukocyte transendothelial migration, our data strongly suggest that paracellular movement of leukocytes into the brain was enhanced by maternal *H. bakeri* infection. In addition to the upregulated leukocyte transendothelial migration pathway, we observed upregulated expression of a variety of integrins that dock leukocytes to endothelial cells. Diapedesis also involves dynamic reshaping of endothelial cells by the actin cytoskeleton^40^ and transient loosening then tightening of tight junctions^41,42^. Gene expression data are consistent with both, as the actin cytoskeleton pathway was upregulated^12^ providing flexibility to the endothelial cells and as genes involved specifically in adheren junctions were upregulated. At first glance, the observed upregulation of junction unit pathways could be interpreted to reflect tightening of the BBB. However, we suggest that, as a response to the junction loosening caused by leukocyte infiltration, upregulated junction expression might restore the selective permeability that is critical for restoring and maintaining BBB integrity.

We had also hypothesized that maternal infection might have influenced transport of immunoglobulin and cytokine signals across the BBB. This transport typically occurs through vesicle mediated endocytosis of receptor-cytokine complexes through the endothelial cells via endosomes^22–24^. The endocytosis pathway was upregulated^12^ as was expression of ligands, receptors, signaling molecules, and endosome formation scissor genes. However, our data indicated that endosome formation was impaired given the downregulation of SNARE interactions for vesicular transport pathway and of several genes related to vesicle formation. This suggests that receptor mediated endocytosis of *H. bakeri* immune markers may not be functional despite it being a common pathway for immune signaling.

A variety of studies over the past decade have begun to detect ways in which GI nematode infections may provide benefits to the infected host^7^ and also to their uninfected offspring^4,8^. It was previously reported that maternal *H. bakeri* infection upregulated expression of LTP and synaptogenesis-related pathways in 7-day old pup brains^12^, pathways that are known to enhance learning and memory^13–15^. Our secondary analysis of gene expression data from these pups also revealed an upregulated Th2 response and upregulated expression of *IL4*, both of which are known to be necessary for memory and learning^43^. Furthermore, as Treg responses promote neural development by mediating axon specification and TGF-β receptor signaling guides neuronal axon initiation in the brain^44^, the observed upregulation of Treg responses would also have potentially positive impacts on learning and memory for the neonate of infected dams. An upregulated Treg response also plays an important role in dampening Th1 inflammation^45^ which would limit neuro-inflammation that in turn compromises the integrity of the BBB^46^. Thus, our results indicate that maternal infection might benefit the neonate by limiting neuro-inflammation and promoting a Th2/Treg environment that might stimulate learning and memory. Similar intergenerational findings may also be found for other maternal infections that induce a Th2/Treg response in the infected host.

A strength of this study was the identification of patterns of differential gene expression within KEGG pathway maps that are not evident by the previously published KEGG pathway analysis alone^12^. As the KEGG pathway analysis relies more on numeric evaluation of gene expression than on a logical analysis based on gene functionality, the results of KEGG pathway analysis can provide contradictory information whereby a pathway can be both upregulated and downregulated simultaneously. Pathway maps typically include three regions: pathway activation mediated by ligands and receptors, propagation of a signal, and formation of products that perform the function of the pathway. As signaling molecules and intermediates are highly redundant and shared among many pathways, our context dependent approach focused on ligands and receptors to assess pathway activation and on products to assess function. To minimize design bias, our search strategy included all possible immune-related genes taken from KEGG pathway maps and from a list of immune-related categories and processes identified from the literature. To lower false discovery rate, more stringent cut-offs for *P*-value and log 2 fold change were used than in the original gene expression database^12^. To minimize confirmation bias, we used every opportunity to receive critiques on the logic of our arguments. To ensure compatibility and consistency, comparisons were made with the few analogous studies. As a result, our approach overcame the limitation of relying only on KEGG pathway analysis and provided internally coherent observations that were consistent with the literature. Nevertheless, we acknowledge that we may have excluded important genes or included genes whose differential expression was of little functional importance. We also acknowledge the limitations associated with reliance on gene expression data with qPCR confirmation of only a few genes and without assaying protein concentrations or conducting functional assays to examine phenotypic effects.

In conclusion, our context relevant interrogation of gene expression in the neonatal brain indicated that a maternal *H. bakeri* infection might promote transendothelial migration of Th2/Treg cells across the BBB of the uninfected neonate and might induce a Th2/Treg response in the neonatal brain. As a Th2/Treg response could have potential benefits in reducing neuro-inflammation and promoting learning and memory, follow-up experimental studies to confirm the gene expression data and to explore neuro-immune development and behavioural responses in the pups of infected dams would be important.

## Methodology

### Source of data

This study was a secondary analysis of immune and BBB related genes that were differentially expressed in the neonatal brain in response to maternal *H. bakeri* infection (https://www.nature.com/articles/s41598-019-40729-w#Sec2)^12^. The original experiment used timed pregnant CD1 outbred mice that had been given a repeated (trickle) infection of 100 ± 3 L3 larvae of *H. bakeri* or a sham infection of distilled water through oral gavage on embryonic days E7, E12, E17, and postpartum day 3 (P3). The trickle infection protocol mimics natural transmission^47^ and allows both larvae and adults to be present simultaneously. Thus the immunoregulation induced by adult worms in the lumen is countered by the immunogenicity of the L4 larvae in the serosal musculature^18^. Pup brains were genotyped on P7 when most neurons have established synaptic connections and during the critical period of synaptogenesis^48,49^. Total brain RNA from one randomly selected male pup per litter (n = 5 per group) was sequenced in an Illumina HiSeq2000 sequencer. The sequence files were analysed using HT-seq^50^ and *NetworkAnalyst*^51^ to identify genes in the pup brain that were differentially expressed in response to maternal nematode infection with adjusted *p* value < 0.05 and log 2 fold change > 1. The original exploration of the KEGG pathway database in *NetworkAnalyst*^12^ provided a list of the differentially expressed pathways with biological significance.

### Procedures for secondary analysis of differentially expressed genes in KEGG pathway maps

For our secondary analysis, we applied more stringent *p* value (< E−5) and log 2 fold change (> 1.2) cut-offs for differential gene expression than had been used in the original analysis^12^ to lower the false recovery rate^52^.

**Figure 1:**
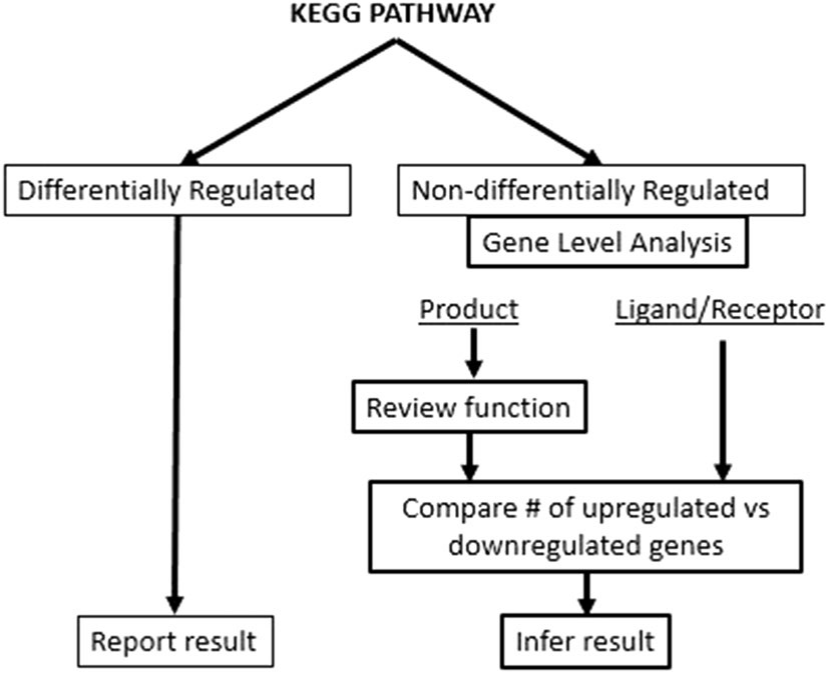
Schematic showing the approach for exploring KEGG pathway maps using the database of differentially expressed genes^12^.

We matched this more stringent gene expression database against genes in KEGG pathway maps (Fig. 1). We focused on the ligand and receptor coding genes that activate the pathway and genes that code for final products rather than intermediates and signaling molecules that have high biological redundancy among pathways and less internal consistency in cascade expression. This allowed us to infer the implications of changes in gene expression for context relevant functions that occur within pathways and that may have been independent of overall differential expression of the KEGG pathway.

### Selection of immune system genes and related KEGG pathways

A list of differentially expressed immune system related genes was created using the more stringent cut-offs (Supplementary Table 1) in order to explore evidence that maternal infection altered expression of the different molecules and cells of the immune system based both on general categories of immune cells and molecules and on genes in immune KEGG pathways.

First, the original database was mined for genes using the categories of cells and molecules involved in any immune response. Cells explored included myeloid cell lineage for innate immune cells (monocyte, macrophage, microglia, dendritic cell, granulocyte, neutrophil, basophil, eosinophil, mast cell) and lymphoid cell lineage for adaptive immune cells (NK cell, lymphoid cell, lymphocyte, T cell, B cell, plasma cell, and leukocyte). Molecules explored included monocyte chemoattractant protein (MCP), colony stimulating factor (CSF), interferon (INF), interleukin (IL), chemokine, immunoglobulin (Ig), tumor necrosis factor (TNF), transforming growth factor (TGF), lymphotoxin, toll-like receptors, CD antigens, major histocompatibility complex (MHC), and selectin. Differential expression of genes in these categories provided the first insight into possible alterations to the immune system in the pup brain in response to maternal infection.

Second, we prepared a list of differentially expressed genes related to the immune system from each of the 21 immune related KEGG pathways (Table 2) (https://www.kegg.jp/kegg/pathway.html) regardless of whether or not they were reported as differentially regulated^12^. The list included inducible proteins, linkers for activation, subunits, inhibitors, activators, receptors, domains, binding proteins, related proteins, “like” genes, other members of the family, and all other intermediate molecules in the pathway.

### Selection of blood brain barrier genes and related KEGG pathways

To determine whether mechanisms known to transport immune cells, cytokines, and immunoglobulins across the BBB were influenced by maternal infection, we made a list of all relevant genes from the KEGG pathway maps for endocytosis and leukocyte transendothelial cell migration. This list included cell adhesion molecules, junction proteins, ligands and receptors, and vesicle formation genes.

### Validation of brain gene expression data

To validate the brain gene expression data obtained from the Illumina Hi-seq sequencing we performed real-time qPCR analysis of three representative genes (*TGFB2, ITGA11* and *VPS29*) following the MIQE guidelines^53^. Already validated primer sequences were obtained from PrimerBank^54^ and purchased from Integrated DNA technologies (Supplementary Table 3). We used frozen whole brain RNA samples from the original experiment^12^ and taking 5ug of total RNA from five pups from control and infection group respectively, we synthesized cDNA using the iScript cDNA Synthesis kit (Bio-Rad, Canada) following instructions from the manufacturer. cDNAs were diluted (1:50) and used for qPCR in a CFX384 (BioRad) machine with the following protocol: initial denaturation at 95 °C for 3 min followed by 39 cycles at 95 °C for 15 s and 60 °C for 45 s for annealing, and finally 95 °C for 10 s. The data were normalized to the geometric mean expression levels of four reference genes (*GADPH, L19, B2M*, and *SDHA*).

## Supplementary Information

Supplementary Tables.

## References

[1] Awasthi S, Bundy D (2007). Intestinal nematode infection and anaemia in developing countries. Br. Med. J..

[2] Odiere MR, Koski KG, Weiler HA, Scott ME (2010). Concurrent nematode infection and pregnancy induce physiological responses that impair linear growth in the murine foetus. Parasitology.

[3] Odiere MR, Scott ME, Leroux L-P, Dzierszinski FS, Koski KG (2012). Maternal protein deficiency during a gastrointestinal nematode infection alters developmental profile of lymphocyte populations and selected cytokines in neonatal mice. J. Nutr..

[4] Darby MG (2019). Pre-conception maternal helminth infection transfers via nursing long-lasting cellular immunity against helminths to offspring. Sci. Adv..

[5] Le Doare K, Holder B, Bassett A, Pannaraj PS (2018). Mother’s milk: A purposeful contribution to the development of the infant microbiota and immunity. Front. Immunol..

[6] Masters S, Barrett-Connor E (1985). Parasites and asthma–predictive or protective?. Epidemiol. Rev..

[7] Ponte EV (2014). Reduced asthma morbidity in endemic areas for helminth infections: A longitudinal ecological study in Brazil. J. Asthma.

[8] Straubinger K (2014). Maternal immune response to helminth infection during pregnancy determines offspring susceptibility to allergic airway inflammation. J. Allergy Clin. Immunol..

[9] Lazarski CA, Ford J, Katzman SD, Rosenberg AF, Fowell DJ (2013). IL-4 attenuates Th1-associated chemokine expression and Th1 trafficking to inflamed tissues and limits pathogen clearance. PLoS ONE.

[10] Lee GR (2018). The balance of Th17 versus Treg cells in autoimmunity. Int. J. Mol. Sci..

[11] Haque M, Starr LM, Koski KG, Scott ME (2018). Differential expression of genes in fetal brain as a consequence of maternal protein deficiency and nematode infection. Int. J. Parasitol..

[12] Haque M, Koski KG, Scott ME (2019). Maternal gastrointestinal nematode infection up-regulates expression of genes associated with long-term potentiation in perinatal brains of uninfected developing pups. Sci. Rep..

[13] Abraham WC, Jones OD, Glanzman DL (2019). Is plasticity of synapses the mechanism of long-term memory storage?. NPJ Sci. Learn..

[14] Stuchlik A (2014). Dynamic learning and memory, synaptic plasticity and neurogenesis: an update. Front. Behav. Neurosci..

[15] Martinez JL, Derrick BE (1996). Long-term potentiation and learning. Annu. Rev. Psychol..

[16] Gadani SP, Cronk JC, Norris GT, Kipnis J (2012). IL-4 in the brain: A cytokine to remember. J. Immunol..

[17] Derecki NC (2010). Regulation of learning and memory by meningeal immunity: A key role for IL-4. J. Exp. Med..

[18] Maizels RM (2012). Immune modulation and modulators in *Heligmosomoides polygyrus* infection. Exp. Parasitol..

[19] Smith MW, Gumbleton M (2006). Endocytosis at the blood-brain barrier: From basic understanding to drug delivery strategies. J. Drug Target.

[20] Carman CV (2009). Mechanisms for transcellular diapedesis: Probing and pathfinding by ‘invadosome-like protrusions’. J. Cell Sci..

[21] Field CJ (2005). The immunological components of human milk and their effect on immune development in infants. J. Nutr..

[22] Pan W (2011). Cytokine signaling modulates blood–brain barrier function. Curr. Pharm. Des..

[23] Filippi M-D (2016). Mechanism of diapedesis: Importance of the transcellular route. Adv. Immunol..

[24] Gao H, Shi W, Freund LB (2005). Mechanics of receptor-mediated endocytosis. Proc. Natl. Acad. Sci. USA.

[25] Bishara, N. The use of biomarkers for detection of early- and late-onset neonatal sepsis. In *Hematology, Immunology and Infectious Disease: Neonatology Questions and Controversies* (Second Edition) (eds. Ohls, R. K. & Maheshwari, A.) 303–315 (W.B. Saunders, 2012).

[26] Schnoor M (2015). Endothelial actin-binding proteins and actin dynamics in leukocyte transendothelial migration. J. Immunol..

[27] Muller WA (2011). Mechanisms of leukocyte transendothelial migration. Annu. Rev. Pathol..

[28] Shen L (2012). Tight junctions on the move: Molecular mechanisms for epithelial barrier regulation. Ann. NY Acad. Sci..

[29] Mellett M (2015). Orphan receptor IL-17RD regulates Toll-like receptor signalling via SEFIR/TIR interactions. Nat. Commun..

[30] Shanks N (2000). Early-life exposure to endotoxin alters hypothalamic-pituitary-adrenal function and predisposition to inflammation. Proc. Natl. Acad. Sci. USA.

[31] Reyes TM, Coe CL (1997). Prenatal manipulations reduce the proinflammatory response to a cytokine challenge in juvenile monkeys. Brain Res..

[32] Shanks N, Larocque S, Meaney MJ (1995). Neonatal endotoxin exposure alters the development of the hypothalamic-pituitary-adrenal axis: Early illness and later responsivity to stress. J. Neurosci..

[33] Shanks N, Lightman SL (2001). The maternal-neonatal neuro-immune interface: Are there long-term implications for inflammatory or stress-related disease?. J. Clin. Invest..

[34] Aarli JA (1983). The immune system and the nervous system. J. Neurol..

[35] Loo Y-M, Gale M (2011). Immune signaling by RIG-I-like receptors. Immunity.

[36] Nakayama T (2017). Th2 cells in health and disease. Annu. Rev. Immunol..

[37] Bao K, Reinhardt RL (2015). The differential expression of IL-4 and IL-13 and its impact on type-2 immunity. Cytokine.

[38] Maddur MS, Bayry J (2015). B cells drive Th2 responses by instructing human dendritic cell maturation. Oncoimmunology..

[39] Harris NL (2006). Mechanisms of neonatal mucosal antibody protection. J. Immunol..

[40] Prasain N, Stevens T (2009). The actin cytoskeleton in endothelial cell phenotypes. Microvasc. Res..

[41] Cavey M, Lecuit T (2009). Molecular bases of cell-cell junctions stability and dynamics. Cold Spring Harb. Perspect. Biol..

[42] Weber CR (2012). Dynamic properties of the tight junction barrier. Ann. NY Acad. Sci..

[43] Brynskikh A, Warren T, Zhu J, Kipnis J (2008). Adaptive immunity affects learning behavior in mice. Brain Behav. Immun..

[44] Yi JJ, Barnes AP, Hand R, Polleux F, Ehlers MD (2010). TGF-β signaling specifies axons during brain development. Cell.

[45] Littringer K (2018). Common features of regulatory T cell specialization during Th1 responses. Front. Immunol..

[46] Kim SY, Buckwalter M, Soreq H, Vezzani A, Kaufer D (2012). Blood-brain barrier dysfunction-induced inflammatory signaling in brain pathology and epileptogenesis. Epilepsia.

[47] Brailsford TJ, Behnke JM (1992). The dynamics of trickle infections with *Heligmosomoides polygyrus* in syngeneic strains of mice. Int. J. Parasitol..

[48] Han X (2009). Transcriptome of embryonic and neonatal mouse cortex by high-throughput RNA sequencing. Proc. Natl. Acad. Sci. USA.

[49] Semple BD (2013). Brain development in rodents and humans: Identifying benchmarks of maturation and vulnerability to injury across species. Prog. Neurobiol..

[50] Anders S, Pyl PT, Huber W (2015). HTSeq—A python framework to work with high-throughput sequencing data. Bioinformatics.

[51] Xia J, Gill EE, Hancock REW (2015). NetworkAnalyst for statistical, visual and network-based meta-analysis of gene expression data. Nat. Protoc..

[52] McCarthy DJ, Smyth GK (2009). Testing significance relative to a fold-change threshold is a TREAT. Bioinformatics.

[53] Bustin SA (2009). The MIQE guidelines: Minimum information for publication of quantitative real-time PCR experiments. Clin. Chem..

[54] Wang, X., Spandidos, A., Wang, H., & Seed, B. PrimerBank: A PCR primer database for quantitative gene expression analysis, 2012 update. Nucleic Acids Res. **40**(Database issue), D1144–9. 10.1093/nar/gkr1013 (2012). 10.1093/nar/gkr1013 PMC3245149 22086960

