# Supplementary material for: Maternal nematode infection upregulates expression of Th2/Treg and diapedesis related genes in the neonatal brain
Source: Sci Rep. 2021 Nov 11;11:22082. doi: 10.1038/s41598-021-01510-0 (PMC8585879; doi:10.1038/s41598-021-01510-0)
Supplement: Supplementary file 1 — Supplementary Tables. [file 41598_2021_1510_MOESM1_ESM.pdf]

Maternal Nematode Infection Upregulates Expression of Th2/Treg and Diapedesis Related Genes In The Neonatal Brain

Nawal El Ahdab[1], Manjurul Haque[1], Ejimedo Madogwe[2], Kristine G. Koski[3] & Marilyn E. Scott[1,4]

[1]Institute of Parasitology, McGill University (Macdonald Campus), 21,111 Lakeshore Road, Ste-Anne de Bellevue, Quebec H9X 3V9, Canada

[2] Department of Animal Science, McGill University (Macdonald Campus), 21,111 Lakeshore Road, Ste-Anne de Bellevue, Quebec H9X 3V9, Canada

[3]School of Human Nutrition, McGill University (Macdonald Campus), 21,111 Lakeshore Road, Ste-Anne de Bellevue, Quebec H9X 3V9, Canada

[4]Correspondence to marilyn.scott@mcgill.ca, Marilyn E. Scott

Supplementary Tables

Supplementary Table 1. Subset of differentially expressed genes that meet the cut-offs of *P* value <E-5 and log fold change >1.2[1].

Gene Name	Gene Symbol	*P*-value	Log 2 fold change
myeloid/lymphoid or mixed-lineage leukemia 1	*MLL1*	6.45E-16	2.6558
myeloid/lymphoid or mixed-lineage leukemia 3	*MLL3*	9.73E-16	1.8857
CDK5 regulatory subunit associated protein 2	*CDK5RAP2*	2.91E-15	1.6542
myeloid/lymphoid or mixed-lineage leukemia 2	*MLL2*	5.27E-15	2.9443
cerebellar degeneration related antigen 1	*CDR1*	1.36E-14	2.6173
RAR-related orphan receptor alpha	*RORA*	1.60E-14	1.6809
nuclear factor of activated T cells 5	*NFAT5*	1.68E-14	2.378
mitogen-activated protein kinase kinase kinase 1	*MAP3K1*	3.18E-14	1.5322
Notch gene homolog 2 (Drosophila)	*NOTCH2*	4.88E-14	2.0131
triggering receptor expressed on myeloid cells 2	*TREM2*	5.35E-14	-1.9824
immunoglobulin superfamily, member 10	*IGSF10*	8.99E-14	2.0756
immunoglobulin superfamily, member 9B	*IGSF9B*	7.18E-13	2.8245
chemokine (C-C motif) ligand 9	*CCL9*	8.11E-13	-2.2198
CREB binding protein	*CREBBP*	1.01E-12	1.8606
coiled-coil domain containing 107	*CCDC107*	1.15E-12	-2.111
nuclear factor I/B	*NFIB*	2.50E-12	1.4485
cystatin B	*CSTB*	2.75E-12	-1.8201
cytochrome c oxidase subunit VIIb	*COX7B*	2.99E-12	-2.2606
RAS-related C3 botulinum substrate 3	*RAC3*	3.28E-12	-1.6253
nuclear factor I/A	*NFIA*	3.48E-12	1.8779
hypoxia inducible factor 3, alpha subunit	*HIF3A*	3.65E-12	1.864
cathepsin H	*CTSH*	4.90E-12	-1.6639
CD302 antigen	*CD302*	7.17E-12	-1.8988
mastermind like 3 (Drosophila)	*MAML3*	7.88E-12	1.6855
ubiquitin specific peptidase 31	*USP31*	1.06E-11	1.8424
mitogen-activated protein kinase kinase kinase 2	*MAP3K2*	1.54E-11	1.368
cytochrome c oxidase subunit IV isoform 1	*COX4I1*	1.58E-11	-2.0354
cytotoxic T lymphocyte-associated protein 2 alpha	*CTLA2A*	1.71E-11	-1.9618
cytochrome c oxidase, subunit VIIc	*COX7C*	2.07E-11	-2.0345
coiled-coil domain containing 32	*CCDC32*	3.27E-11	-1.6673
cytochrome c oxidase, subunit VIIa 2	*COX7A2*	3.53E-11	-1.9814
CD320 antigen	*CD320*	3.60E-11	-1.3617
S100 calcium binding protein A10 (calpactin)	*S100A10*	5.70E-11	-1.783
integrin beta 1 binding protein 1	*ITGB1BP1*	6.09E-11	-1.9182
guanine nucleotide binding protein (G protein), beta polypeptide 2 like 1	*GNB2L1*	6.33E-11	-1.8365
nuclear receptor co-repressor 2	*NCOR2*	6.36E-11	2.4817
coiled-coil domain containing 72	*CCDC72*	6.73E-11	-1.824
late endosomal/lysosomal adaptor, MAPK and MTOR activator 3	*LAMTOR3*	8.47E-11	-1.5656
cytochrome c oxidase, subunit VIb polypeptide 1	*COX6B1*	8.85E-11	-1.8064
BCL2-associated agonist of cell death	*BAD*	9.98E-11	-1.5347
BCL2-like 11 (apoptosis facilitator)	*BCL2L11*	1.10E-10	1.3264
a disintegrin-like and metallopeptidase (reprolysin type) with thrombospondin type 1 motif, 1	*ADAMTS1*	1.12E-10	1.3939
coiled-coil domain containing 56	*CCDC56*	1.19E-10	-1.8499

forkhead box N3	*FOXN3*	1.21E-10	1.7673
chemokine (C-C motif) ligand 6	*CCL6*	1.49E-10	-1.9152
coiled-coil domain containing 153	*CCDC153*	1.60E-10	-1.8643
RAS-like, family 11, member A	*RASL11A*	1.76E-10	-1.535
cytochrome c oxidase, subunit VIc	*COX6C*	1.89E-10	-1.9396
CD53 antigen	*CD53*	1.94E-10	-1.5542
ubiquitin specific peptidase 34	*USP34*	1.94E-10	1.5945
nuclear factor I/C	*NFIC*	1.94E-10	1.9803
mitogen-activated protein kinase kinase kinase 13	*MAP3K13*	2.04E-10	2.2629
GATA zinc finger domain containing 2B	*GATAD2B*	2.28E-10	1.8123
natural killer tumor recognition sequence	*NKTR*	2.50E-10	1.2598
src homology 2 domain-containing transforming protein C3	*SHC3*	2.52E-10	2.036
CUGBP, Elav-like family member 2	*CELF2*	2.56E-10	1.8558
immunoglobulin superfamily, DCC subclass, member 4	*IGDCC4*	2.63E-10	1.6464
lymphocyte protein tyrosine kinase	*LCK*	3.31E-10	-1.2848
interleukin-1 receptor-associated kinase 1 binding protein 1	*IRAK1BP1*	3.38E-10	-1.5769
BCL2/adenovirus E1B interacting protein 3	*BNIP3*	4.27E-10	-1.6971
CD40 antigen	*CD40*	4.33E-10	-1.7484
anaphase promoting complex subunit 13	*ANAPC13*	4.39E-10	-1.7916
interleukin 17 receptor D	*IL17RD*	4.41E-10	1.9374
B cell leukemia/lymphoma 2	*BCL2*	4.84E-10	1.3446
mastermind like 2 (Drosophila)	*MAML2*	5.19E-10	1.4874
anaphase promoting complex subunit 11	*ANAPC11*	5.24E-10	-1.6355
ras responsive element binding protein 1	*RREB1*	5.37E-10	2.0917
protein kinase, interferon inducible double stranded RNA dependent activator	*PRKRA*	6.72E-10	-1.4743
retinoic acid receptor responder (tazarotene induced) 2	*RARRES2*	6.81E-10	-1.5707
B cell leukemia/lymphoma 2 related protein A1b	*BCL2A1B*	7.66E-10	-2.9945
histone deacetylase 4	*HDAC4*	8.23E-10	1.8345
splicing factor 3b, subunit 5	*SF3B5*	8.72E-10	-1.5446
myeloid/lymphoid or mixed-lineage leukemia 5	*MLL5*	8.74E-10	1.9847
coiled-coil domain containing 90B	*CCDC90B*	9.27E-10	-1.6183
BCL2/adenovirus E1B interacting protein 1	*BNIP1*	1.10E-09	-1.3242
coiled-coil domain containing 23	*CCDC23*	1.14E-09	-1.3368
a disintegrin-like and metallopeptidase (reprolysin type) with thrombospondin type 1 motif, 9	*ADAMTS9*	1.38E-09	1.321
interleukin 13 receptor, alpha 2	*IL13RA2*	1.62E-09	-1.9619
nuclear receptor co-repressor 1	*NCOR1*	1.66E-09	1.5845
a disintegrin-like and metallopeptidase (reprolysin type) with thrombospondin type 1 motif, 12	*ADAMTS12*	1.68E-09	1.9621
RAB7, member RAS oncogene family-like 1	*RAB7L1*	1.71E-09	-1.4527
integrin beta 4	*ITGB4*	1.71E-09	1.8016
RAS related protein 1b	*RAP1B*	1.78E-09	-1.5287
forkhead box O3	*FOXO3*	1.87E-09	1.7157
TNF receptor-associated factor 3	*TRAF3*	1.91E-09	2.0585
CD9 antigen	*CD9*	2.11E-09	-1.6197
glycogen synthase kinase 3 beta	*GSK3B*	2.22E-09	1.9234
interferon, alpha-inducible protein 27 like 2A	*IFI27L2A*	2.51E-09	-2.5516

cytochrome c oxidase, subunit VI a, polypeptide 1	*COX6A1*	2.67E-09	-1.4456
TRAF-interacting protein with forkhead-associated domain	*TIFA*	2.81E-09	-1.6203
coiled-coil domain containing 53	*CCDC53*	2.87E-09	-1.4254
RAB26, member RAS oncogene family	*RAB26*	3.00E-09	-1.5025
a disintegrin-like and metallopeptidase (reprolysin type) with thrombospondin type 1 motif, 18	*ADAMTS18*	3.13E-09	1.2523
MAD homolog 9 (Drosophila)	*SMAD9*	3.38E-09	1.3487
cytochrome c oxidase, subunit Vb	*COX5B*	3.57E-09	-1.5843
cytotoxic T lymphocyte-associated protein 2 beta	*CTLA2B*	3.60E-09	-2.041
COX16 cytochrome c oxidase assembly homolog (S. cerevisiae)	*COX16*	4.15E-09	-1.7752
chemokine (C-C motif) ligand 27A	*CCL27A*	4.27E-09	-1.8182
dedicator of cytokinesis 1	*DOCK1*	4.35E-09	1.4669
coiled coil domain containing 28B	*CCDC28B*	4.48E-09	-1.2477
interleukin 18	*IL18*	4.49E-09	-1.4295
CDC42 binding protein kinase alpha	*CDC42BPA*	4.60E-09	2.2741
cytochrome c oxidase, subunit VIIIa	*COX8A*	5.02E-09	-1.3628
phosphatidylinositol 3-kinase, regulatory subunit, polypeptide 1 (p85 alpha)	*PIK3R1*	5.40E-09	1.3438
malignant T cell amplified sequence 1	*MCTS1*	5.53E-09	-1.339
ataxin 1	*ATXN1*	5.65E-09	1.951
mitogen-activated protein kinase kinase kinase 9	*MAP3K9*	6.19E-09	2.4578
BCL6 co-repressor-like 1	*BCORL1*	6.45E-09	1.8165
coiled-coil domain containing 88C	*CCDC88C*	6.79E-09	1.8684
nuclear factor of activated T cells, cytoplasmic, calcineurin dependent 3	*NFATC3*	6.84E-09	1.2199
BCL6 interacting corepressor	*BCOR*	6.95E-09	1.3748
myeloid/lymphoid or mixed-lineage leukemia (trithorax homolog, Drosophila); translocated to, 4	*MLLT4*	7.29E-09	1.5566
ADAMTS-like 1	*ADAMTSL1*	7.38E-09	1.6231
cystatin E/M	*CST6*	7.67E-09	-1.6213
coiled-coil domain containing 115	*CCDC115*	7.91E-09	-1.5257
MAP3K12 binding inhibitory protein 1	*MBIP*	8.55E-09	-1.5337
anaphase promoting complex subunit 10	*ANAPC10*	9.23E-09	-1.5178
coiled-coil domain containing 84	*CCDC84*	1.04E-08	-1.7195
T cell leukemia translocation altered gene	*TCTA*	1.05E-08	-1.4425
RAN, member RAS oncogene family	*RAN*	1.06E-08	-1.4659
adenylate cyclase 1	*ADCY1*	1.13E-08	2.386
ubiquitin specific peptidase 24	*USP24*	1.15E-08	1.3304
PAK1 interacting protein 1	*PAK1IP1*	1.16E-08	-1.2902
BCL2/adenovirus E1B interacting protein 3-like	*BNIP3L*	1.18E-08	-1.3702
non-catalytic region of tyrosine kinase adaptor protein 1	*NCK1*	1.19E-08	-1.3549
sema domain, immunoglobulin domain (Ig), transmembrane domain (TM) and short cytoplasmic domain, (semaphorin) 4D	*SEMA4D*	1.24E-08	1.3942
cytochrome c oxidase, subunit VIIa 1	*COX7A1*	1.28E-08	-2.1301
Ras-like without CAAX 1	*RIT1*	1.35E-08	-1.3489
RAR-related orphan receptor beta	*RORB*	1.36E-08	1.4579
Notch gene homolog 1 (Drosophila)	*NOTCH1*	1.47E-08	2.4114

a disintegrin and metallopeptidase domain 23	*ADAM23*	1.50E-08	1.5141
a disintegrin-like and metallopeptidase (reprolysin type) with thrombospondin type 1 motif, 17	*ADAMTS17*	1.54E-08	1.597
lymphocyte antigen 86	*LY86*	1.55E-08	-1.6468
chemokine (C-C motif) ligand 25	*CCL25*	1.74E-08	-1.6614
a disintegrin and metallopeptidase domain 12 (meltrin alpha)	*ADAM12*	1.79E-08	1.5943
dedicator of cytokinesis 5	*DOCK5*	1.88E-08	1.8291
ubiquitin specific peptidase 49	*USP49*	2.02E-08	1.8333
cell division cycle 26	*CDC26*	2.09E-08	-1.2625
cell division cycle 34 homolog (S. cerevisiae)	*CDC34*	2.10E-08	-1.2529
ubiquitin specific peptidase 36	*USP36*	2.13E-08	1.3243
Wiskott-Aldrich syndrome-like (human)	*WASL*	2.16E-08	1.3013
myeloid leukemia factor 1	*MLF1*	2.18E-08	-1.3263
integrin alpha 11	*ITGA11*	2.20E-08	1.7196
Ras homolog enriched in brain	*RHEB*	2.25E-08	-1.281
S100 calcium binding protein A16	*S100A16*	2.28E-08	-1.4785
son of sevenless homolog 1 (Drosophila)	*SOS1*	2.33E-08	1.4037
tumor necrosis factor receptor superfamily, member 11a	*TNFRSF11A*	2.39E-08	1.2215
CDC42 binding protein kinase gamma (DMPK-like)	*CDC42BPG*	2.45E-08	1.5903
retinoid X receptor alpha	*RXRA*	2.64E-08	1.4956
RAB9, member RAS oncogene family	*RAB9*	2.66E-08	-1.4424
immunoglobulin superfamily, member 3	*IGSF3*	2.69E-08	1.7284
microtubule-associated protein 1 light chain 3 beta	*MAP1LC3B*	2.73E-08	-1.2518
T-box 20	*TBX20*	2.81E-08	2.5761
mechanistic target of rapamycin (serine/threonine kinase)	*MTOR*	2.95E-08	1.3607
CD83 antigen	*CD83*	3.03E-08	-1.2487
S100 calcium binding protein A8 (calgranulin A)	*S100A8*	3.07E-08	-2.4356
anaphase promoting complex subunit 16	*ANAPC16*	3.07E-08	-1.3616
leukocyte specific transcript 1	*LST1*	3.30E-08	-1.4504
a disintegrin-like and metallopeptidase (reprolysin type) with thrombospondin type 1 motif, 15	*ADAMTS15*	3.37E-08	1.4456
coiled-coil domain containing 58	*CCDC58*	3.47E-08	-1.4715
integrin alpha 4	*ITGA4*	3.81E-08	1.2737
mastermind-like domain containing 1	*MAMLD1*	4.00E-08	1.4065
CD300A antigen	*CD300A*	4.65E-08	-1.4979
a disintegrin-like and metallopeptidase (reprolysin type) with thrombospondin type 1 motif, 3	*ADAMTS3*	4.67E-08	1.5286
S100 calcium binding protein A1	*S100A1*	4.68E-08	-1.6934
transforming growth factor, beta receptor III	*TGFBR3*	4.69E-08	1.2645
CD59a antigen	*CD59A*	4.72E-08	-1.7165
chemokine (C-X-C motif) ligand 1	*CXCL1*	5.24E-08	-1.9013
myeloid/lymphoid or mixed-lineage leukemia (trithorax homolog, Drosophila); translocated to, 6	*MLLT6*	5.54E-08	1.4785
tumor necrosis factor, alpha-induced protein 8-like 2	*TNFAIP8L2*	5.60E-08	-1.6267
mastermind like 1 (Drosophila)	*MAML1*	5.77E-08	1.5213
forkhead box K1	*FOXK1*	5.90E-08	2.163
tolloid-like	*TLL1*	6.17E-08	1.4164
chemokine (C-C motif) receptor 1	*CCR1*	6.32E-08	-1.5864
MAD homolog 3 (Drosophila)	*SMAD3*	6.35E-08	1.6784

cytochrome c oxidase subunit III	*COX3*	6.77E-08	-1.3201
interleukin enhancer binding factor 2	*ILF2*	6.79E-08	-1.2226
COX19 cytochrome c oxidase assembly homolog (S. cerevisiae)	*COX19*	7.56E-08	-1.2507
Ras association (RalGDS/AF-6) domain family (N-terminal) member 7	*RASSF7*	7.57E-08	-1.2105
adenylate cyclase 9	*ADCY9*	7.78E-08	1.978
CD63 antigen	*CD63*	9.54E-08	-1.4596
forkhead box O1	*FOXO1*	9.61E-08	1.7171
a disintegrin and metallopeptidase domain 19 (meltrin beta)	*ADAM19*	9.92E-08	1.7922
CDC-like kinase 1	*CLK1*	1.03E-07	-1.389
JNK1/MAPK8-associated membrane protein	*JKAMP*	1.03E-07	-1.2537
arrestin, beta 1	*ARRB1*	1.09E-07	1.8425
runt-related transcription factor 1; translocated to, 1 (cyclin D-related)	*RUNX1T1*	1.14E-07	1.4627
dedicator of cyto-kinesis 3	*DOCK3*	1.23E-07	1.9443
interleukin 1 beta	*IL1B*	1.33E-07	-3.1953
latent transforming growth factor beta binding protein 3	*LTBP3*	1.35E-07	1.3637
adaptor protein complex AP-1, mu 2 subunit	*AP1M2*	1.38E-07	-1.3653
ADAMTS-like 3	*ADAMTSL3*	1.38E-07	1.3184
guanine nucleotide binding protein, alpha q polypeptide	*GNAQ*	1.43E-07	1.5085
phosphoinositide-3-kinase, class 2, beta polypeptide	*PIK3C2B*	1.54E-07	1.7893
ubiquitin specific peptidase 13 (isopeptidase T-3)	*USP13*	1.54E-07	1.2968
cystatin C	*CST3*	1.55E-07	-1.4457
immunoglobulin-like domain containing receptor 2	*ILDR2*	1.57E-07	1.2626
forkhead box J2	*FOXJ2*	1.61E-07	1.5213
Notch gene homolog 3 (Drosophila)	*NOTCH3*	1.62E-07	2.1223
discs, large (Drosophila) homolog-associated protein 1	*DLGAP1*	1.64E-07	1.5008
p21 protein (Cdc42/Rac)-activated kinase 4	*PAK4*	1.78E-07	1.4325
interleukin 4	*IL4*	1.81E-07	1.2171
a disintegrin-like and metallopeptidase (reprolysin type) with thrombospondin type 1 motif, 7	*ADAMTS7*	1.87E-07	1.2347
SRC kinase signaling inhibitor 1	*SRCIN1*	2.02E-07	1.8333
CD86 antigen	*CD86*	2.09E-07	-1.4856
Duffy blood group, chemokine receptor	*DARC*	2.20E-07	1.8805
Snf2-related CREBBP activator protein	*SRCAP*	2.21E-07	1.3655
S100 calcium binding protein A9 (calgranulin B)	*S100A9*	2.39E-07	-2.0865
Ras homolog enriched in brain like 1	*RHEBL1*	2.67E-07	-1.22
discs, large (Drosophila) homolog-associated protein 2	*DLGAP2*	2.69E-07	1.7469
dedicator of cytokinesis 9	*DOCK9*	2.86E-07	1.5384
B cell leukemia/lymphoma 11B	*BCL11B*	2.93E-07	2.0642
caspase 1	*CASP1*	3.03E-07	-1.323
protein kinase C, alpha	*PRKCA*	3.03E-07	1.5849
dishevelled 3, dsh homolog (Drosophila)	*DVL3*	3.09E-07	1.8678
CDC28 protein kinase regulatory subunit 2	*CKS2*	3.17E-07	-1.4964
a disintegrin-like and metallopeptidase (reprolysin type) with thrombospondin type 1 motif, 2	*ADAMTS2*	3.18E-07	1.5077
interleukin 10-related T cell-derived inducible factor beta	*ILTIFB*	3.20E-07	-2.3961
T cell lymphoma invasion and metastasis 1	*TIAM1*	3.23E-07	2.0286

discs, large homolog 2 (Drosophila)	*DLG2*	3.24E-07	1.3519
cathepsin O	*CTSO*	3.43E-07	-1.2282
forkhead box J3	*FOXJ3*	3.47E-07	1.2904
transcription factor 7 like 1 (T cell specific, HMG box)	*TCF7L1*	3.83E-07	1.5258
coiled-coil domain containing 6	*CCDC6*	3.94E-07	1.701
RAS-related protein-1a	*RAP1A*	4.09E-07	-1.3125
forkhead box M1	*FOXM1*	4.12E-07	1.3554
B cell CLL/lymphoma 9	*BCL9*	4.40E-07	2.0256
forkhead box P1	*FOXP1*	4.55E-07	1.395
ELAV (embryonic lethal, abnormal vision, Drosophila)-like 3 (Hu antigen C)	*ELAVL3*	4.64E-07	1.5461
Casitas B-lineage lymphoma b	*CBLB*	4.87E-07	1.4681
discs, large homolog 5 (Drosophila)	*DLG5*	4.89E-07	1.7797
fos-like antigen 2	*FOSL2*	5.02E-07	1.6381
CD48 antigen	*CD48*	5.13E-07	-1.8838
coiled-coil domain containing 160	*CCDC160*	5.14E-07	-1.0062
CDC28 protein kinase 1b	*CKS1B*	5.41E-07	-1.305
CD84 antigen	*CD84*	6.02E-07	-1.4359
caspase 12	*CASP12*	6.29E-07	-1.3853
interferon gamma inducible protein 47	*IFI47*	6.30E-07	-1.6666
ubiquitin specific peptidase 42	*USP42*	6.56E-07	1.3803
CUGBP, Elav-like family member 1	*CELF1*	6.56E-07	1.2228
phospholipase C, gamma 1	*PLCG1*	6.82E-07	1.248
chemokine (C-C motif) ligand 12	*CCL12*	7.17E-07	-1.9695
mature T cell proliferation 1	*MTCP1*	7.32E-07	-1.3497
B cell CLL/lymphoma 9-like	*BCL9L*	7.61E-07	2.182
RAB32, member RAS oncogene family	*RAB32*	7.65E-07	-1.2409
interferon-stimulated protein	*ISG20*	7.81E-07	-1.442
mitogen-activated protein kinase binding protein 1	*MAPKBP1*	8.08E-07	1.5018
phospholipase C, beta 1	*PLCB1*	8.37E-07	1.2312
nuclear factor I/X	*NFIX*	8.62E-07	2.0714
a disintegrin and metallopeptidase domain 22	*ADAM22*	8.70E-07	1.2892
TGF-beta activated kinase 1/MAP3K7 binding protein 3	*TAB3*	8.87E-07	1.2923
transforming growth factor, beta receptor associated protein 1	*TGFBRAP1*	8.92E-07	1.3063
CD209f antigen	*CD209F*	9.01E-07	-1.4813
nuclear factor of activated T cells, cytoplasmic, calcineurin dependent 1	*NFATC1*	9.49E-07	1.3363
NCK-associated protein 5	*NCKAP5*	9.89E-07	1.4162
FAD-dependent oxidoreductase domain containing 2	*FOXRED2*	1.02E-06	1.4511
cytochrome c oxidase subunit VIIb2	*COX7B2*	1.04E-06	-1.7261
S100 calcium binding protein A13	*S100A13*	1.11E-06	-1.388
integrin alpha 3	*ITGA3*	1.16E-06	1.2366
coiled-coil domain containing 28A	*CCDC28A*	1.22E-06	-1.297
X-linked lymphocyte-regulated complex	*XLR*	1.26E-06	-1.7171
transforming growth factor alpha	*TGFA*	1.30E-06	1.2669
interleukin 12 receptor, beta 2	*IL12RB2*	1.32E-06	2.0879
hypoxia-inducible factor 1, alpha subunit inhibitor	*HIF1AN*	1.33E-06	1.2766
adenylate cyclase 5	*ADCY5*	1.41E-06	1.7851
adenylate cyclase 6	*ADCY6*	1.42E-06	1.2083

GRB10 interacting GYF protein 1	*GIGYF1*	1.54E-06	1.5862
T-cell immunoglobulin and mucin domain containing 2 pseudogene	*GM4926*	1.69E-06	-1.5855
sema domain, immunoglobulin domain (Ig), short basic domain, secreted, (semaphorin) 3G	*SEMA3G*	1.73E-06	1.424
immunoglobulin superfamily, DCC subclass, member 3	*IGDCC3*	2.05E-06	1.4696
squamous cell carcinoma antigen recognized by T cells 1	*SART1*	2.06E-06	1.2246
interferon-induced protein 35	*IFI35*	2.15E-06	-1.3033
sema domain, immunoglobulin domain (Ig), transmembrane domain (TM) and short cytoplasmic domain, (semaphorin) 4G	*SEMA4G*	2.18E-06	1.5283
S100 calcium binding protein A5	*S100A5*	2.61E-06	-2.8835
NCK-associated protein 5-like	*NCKAP5L*	2.71E-06	1.8654
RAB6B, member RAS oncogene family	*RAB6B*	2.86E-06	1.3191
mitogen-activated protein kinase 8 interacting protein 3	*MAPK8IP3*	2.91E-06	1.5485
mitogen-activated protein kinase kinase kinase 5	*MAP3K5*	2.96E-06	1.269
protein kinase C and casein kinase substrate in neurons 1	*PACSIN1*	3.06E-06	1.4931
MAP/microtubule affinity-regulating kinase 4	*MARK4*	3.17E-06	1.4975
vav 2 oncogene	*VAV2*	3.25E-06	1.4015
chemokine-like factor	*CKLF*	3.37E-06	-1.2909
latent transforming growth factor beta binding protein 4	*LTBP4*	3.43E-06	1.6984
linker for activation of T cells family, member 2	*LAT2*	3.64E-06	-1.9848
coiled-coil domain containing 103	*CCDC103*	4.09E-06	-1.2257
microtubule-associated protein, RP/EB family, member 2	*MAPRE2*	4.57E-06	1.3526
Rous sarcoma oncogene	*SRC*	5.11E-06	1.462
splicing factor 1	*SF1*	5.12E-06	1.3615
myeloid-associated differentiation marker	*MYADM*	5.14E-06	1.2936
discs, large (Drosophila) homolog-associated protein 3	*DLGAP3*	5.51E-06	1.4799
tumor necrosis factor receptor superfamily, member 12a	*TNFRSF12A*	6.00E-06	-1.2305
MAP-kinase activating death domain	*MADD*	6.08E-06	1.2296
interleukin 15	*IL15*	6.30E-06	-1.662
CDC42 binding protein kinase beta	*CDC42BPB*	7.17E-06	1.8055
guanine nucleotide binding protein, alpha O	*GNAO1*	7.35E-06	1.4771
MAP/microtubule affinity-regulating kinase 1	*MARK1*	7.88E-06	1.2378
CCR4-NOT transcription complex, subunit 3	*CNOT3*	8.09E-06	1.2313
interleukin 22	*IL22*	9.41E-06	-2.162
mitogen-activated protein kinase kinase kinase 8	*MAP3K8*	1.01E-05	-1.4099
CD93 antigen	*CD93*	1.05E-05	1.3519
nuclear factor of activated T cells, cytoplasmic, calcineurin dependent 2	*NFATC2*	1.11E-05	1.2136
immunoglobulin superfamily, member 5	*IGSF5*	1.25E-05	-1.2568
Ras and Rab interactor 3	*RIN3*	1.27E-05	1.3482
immunoglobulin superfamily, member 6	*IGSF6*	1.29E-05	-1.3329
protein kinase C, epsilon	*PRKCE*	1.29E-05	1.4301
p21 protein (Cdc42/Rac)-activated kinase 6	*PAK6*	1.37E-05	1.2262
CD52 antigen	*CD52*	1.50E-05	-1.7423
delta-like 1 (Drosophila)	*DLL1*	1.53E-05	1.2633
splicing factor proline/glutamine rich (polypyrimidine tract binding protein associated)	*SFPQ*	1.56E-05	1.2486
CD1d1 antigen	*CD1D1*	1.60E-05	-1.2071

tolloid-like 2	*TLL2*	1.75E-05	1.5856
aryl hydrocarbon receptor nuclear translocator 2	*ARNT2*	1.84E-05	1.2532
deltex 3-like (Drosophila)	*DTX3L*	1.86E-05	1.2808
CD200 receptor 1	*CD200R1*	1.99E-05	-1.5711
phosphatidylinositol-3,4,5-trisphosphate-dependent Rac exchange factor 1	*PREX1*	2.05E-05	1.2251
cytochrome c oxidase, subunit XVII assembly protein homolog (yeast)	*COX17*	2.64E-05	-1.2697
linker for activation of T cells	*LAT*	2.73E-05	-1.9806
hairless	*HR*	2.82E-05	1.4814
forkhead box D3	*FOXD3*	2.86E-05	1.4502
TNF receptor superfamily, member 6	*FAS*	2.91E-05	-1.6839
caspase 4, apoptosis-related cysteine peptidase	*CASP4*	2.97E-05	-1.6619
chemokine (C-C motif) ligand 7	*CCL7*	3.00E-05	-1.6164
T-box 3	*TBX3*	3.09E-05	1.2446
RAB38, member of RAS oncogene family	*RAB38*	3.15E-05	-1.2267
deltex 1 homolog (Drosophila)	*DTX1*	3.64E-05	1.4082
CD209g antigen	*CD209G*	3.77E-05	-1.9848
adenylate cyclase 3	*ADCY3*	3.83E-05	1.2505
matrix metallopeptidase 13	*MMP13*	4.26E-05	-1.7194
chemokine (C-X-C motif) ligand 11	*CXCL11*	4.45E-05	-1.7877
matrix metallopeptidase 15	*MMP15*	4.67E-05	1.295
interleukin 15 receptor, alpha chain	*IL15RA*	4.79E-05	-1.3734
CUGBP, Elav-like family member 5	*CELF5*	4.90E-05	1.3492
chemokine (C-X-C motif) receptor 5	*CXCR5*	6.26E-05	1.7003
retinoic acid receptor, alpha	*RARA*	6.41E-05	1.2092
adaptor protein complex AP-2, alpha 1 subunit	*AP2A1*	6.49E-05	1.3822
RAB17, member RAS oncogene family	*RAB17*	7.18E-05	-2.0473
lymphocyte antigen 6 complex, locus G6D	*LY6G6D*	7.51E-05	-2.0459
cytochrome c oxidase, subunit VI a, polypeptide 2	*COX6A2*	8.74E-05	-1.2031
forkhead box P4	*FOXP4*	9.68E-05	1.2832
chemokine (C-C motif) ligand 24	*CCL24*	9.86E-05	-1.2683

[1] Differential regulation as reported by Haque et al. [12]

Supplementary Table 2. Brain qPCR expression data for 7-day old pups of uninfected dams and of dams infected with *Heligmosomoides bakeri* during pregnancy and lactation. Data normalized to the geometric mean expression of four reference genes (*GADPH, L19, B2M* and *SDHA*).

Gene	Pups of Uninfected Dams	Pups of Infected Dams	P-value
TGFB2	0.31 ± 0.13	0.96 ± 0.05	0.0014
ITGA11	0.38 ± 0.15	0.88 ± 0.06	0.0107
VPS29	1.42 ± 0.28	0.96 ± 0.04	0.1517

Supplementary Table 3. List of primers used in the qPCR validation of gene expression

**Gene**	**Forward primer (5′-3′)**	**Reverse primer (5′-3′)**	**Amplification efficiency (%)**
TGFB2	CTTCGACGTGACAGACGCT	GCAGGGGCAGTGTAAACTTATT	96.7
ITGA11	CCTTCCCTCGGATGTGAGTCA	AAGTTCTCCCCGTATGGTAAGA	95.8
VPS29	CTGCACCAAGGAGAGCTACG	TCAGACCGATCTTGAACTGGC	99.0
GAPDH	AGGTCGGTGTGAACGGATTTG	TGTAGACCATGTAGTTGAGGTCA	98.3
L19	ATGAGTATGCTCAGGCTACAGA	GCATTGGCGATTTCATTGGTC	96.5
B2M	TTCTGGTGCTTGTCTCACTGA	CAGTATGTTCGGCTTCCCATTC	96.6
SDHA	GGAACACTCCAAAAACAGACCT	CCACCACTGGGTATTGAGTAGAA	98.1
